# The independent association of nutritional status with quality of life beyond depression, frailty, and loneliness in community-dwelling older adults: a cross-sectional study

**DOI:** 10.1186/s12877-026-07772-5

**Published:** 2026-07-03

**Authors:** Kadriye Toprak, Makbule Ercakir, Pinar Gobel

**Affiliations:** 1 Department of Nutrition and Dietetics, School of Health Sciences, Ankara Medipol University, Altındağ, Ankara, 06050 Türkiye; 2 Independent Researcher, Çankaya, Ankara, 06690 Türkiye; 3 Department of Nutrition and Dietetics, Gülhane School of Health Sciences, University of Health Science, Keçiören, Ankara, 06010 Türkiye

**Keywords:** Aging, Geriatric, Malnutrition, Quality of life, Depression, Frailty, Loneliness, MNA-SF

## Abstract

**Background:**

This study aimed to assess the nutritional status of community-dwelling older adults and to investigate the unique explanatory power of nutritional status for quality of life, while controlling for psychosocial factors such as depression, frailty, and loneliness.

**Methods:**

This cross-sectional study was conducted with 758 participants (330 men, 428 women; age: 71.1 ± 5.79 years) aged 65 and over living in Ankara. Nutritional status was assessed using the Mini Nutritional Assessment-Short Form (MNA-SF), loneliness perception using the UCLA Loneliness Scale, frailty using the FRAIL Scale, depression using the Geriatric Depression Scale (GDS-15), and quality of life using the EUROHIS-QOL-8. Data were analyzed using multiple linear regression and three-stage hierarchical regression analysis.

**Results:**

Nutritional assessment showed that 34.7% of participants were at risk of malnutrition, and 7.3% were malnourished. Multiple regression analysis identified depression, Body Mass Index (BMI), frailty, loneliness, and the number of comorbidities as significant predictors of nutritional status (*p* < 0.05). The strongest correlation was observed between depression and quality of life (*p* < 0.001). Hierarchical regression indicated that the inclusion of psychosocial variables significantly increased the model’s explanatory power (*p* < 0.001). Notably, MNA-SF scores remained an independent predictor of quality of life (*p* < 0.01) even after controlling for all psychosocial factors. Depression, frailty, and loneliness persisted as significant predictors in the final model (*p* < 0.01).

**Conclusions:**

This study revealed that nutritional status is independently associated with quality of life in older adults, independent of strong psychosocial determinants. Although depression, frailty, and loneliness were dominant predictors, the independent contribution of nutritional status suggests that nutritional assessment and interventions could be considered as an integral part of geriatric care. The findings highlight the potential role of comprehensive care models that combine psychosocial support, mental health services, and nutritional interventions in improving quality of life, subject to further confirmation through interventional studies. These results may inform the consideration of including nutrition, depression, frailty, and loneliness screenings in routine geriatric assessments in primary healthcare settings, pending longitudinal validation.

**Supplementary Information:**

The online version contains supplementary material available at 10.1186/s12877-026-07772-5.

## Background

Globally, demographic structures are rapidly changing, and the older adult population is increasing. With increasing life expectancy, one of the primary goals has become enhancing the quality of aging and promoting healthy aging [1]. Quality of life in older adults is defined as a dynamic, multidimensional interaction among physical health, psychological well-being, social relationships, and environmental factors. Evidence from community-based research suggests that QoL is not only a reflection of health status but is also significantly shaped by age, socioeconomic status, and chronic disease burden [2, 3].

Malnutrition is considered a significant preventable risk factor associated with a decline in the quality of life among older adults [1]. Malnutrition is a comprehensive geriatric syndrome involving decreased energy intake and impaired functional capacity [4]. The literature shows that the prevalence of malnutrition varies widely depending on geographic regions, socioeconomic status, and the screening tools used [4, 5]. Similarly, recent studies in Türkiye indicate that, although the screening methods vary, malnutrition remains a widespread and serious public health issue among the older adults [6, 7]. Given that Türkiye is experiencing one of the fastest population aging rates globally, understanding these dynamics in a metropolitan context like Ankara is crucial. In this setting, the rapid transition from traditional extended families to nuclear structures may further exacerbate the link of psychosocial factors like loneliness with nutritional health [7]. Rather than arising from a single factor, the development of malnutrition is a complex process where multiple chronic diseases and polypharmacy converge to disrupt appetite regulation [8]. Current diagnostic methods, especially the GLIM (Global Leadership Initiative on Malnutrition) criteria, further support the strong association between malnutrition, the burden of chronic disease, and overall health perception [9].

Nutritional status also interacts strongly with physical frailty, a syndrome characterized by decreased physiological capacity [10]. These two conditions frequently coexist, creating a reciprocal cycle where malnutrition is associated with physical frailty through fatigue and sarcopenia, while frailty is associated with functional dependence [10, 11]. Studies highlight the increased prevalence of muscle wasting in older adults at nutritional risk, accelerating the frailty process and making them more vulnerable [12–14]. The combination of these two conditions is linked to significantly higher rates of adverse health outcomes and mortality risk than either alone [15]. These findings collectively underscore the synergistic relationship between nutritional decline and physical vulnerability [10–15].

In addition to physical factors, psychosocial factors also play a significant role in this process. Depression, common among the older adults, is a strong risk factor for malnutrition associated with reduced food intake through anhedonia [9, 16]. Similarly, loneliness and social isolation are often associated with a loss of the social eating experience, reducing appetite and decreasing the quality of meals consumed [17]. Longitudinal studies have shown that older adults who feel lonely experience a faster decline in quality of life over time and are at increased nutritional risk [18, 19]. It is reported that social isolation, depression, and nutritional deficiencies form a triple risk cluster that threatens an individual’s life satisfaction and cognitive functions [19]. It is also stated that this psychological burden associated with frailty and malnutrition may be significantly associated with the quality of life in older adults and linked to serious consequences such as hopelessness and suicidal tendencies [20]. However, most existing studies examine these factors in isolation, which limits a comprehensive understanding of how psychosocial variables collectively relate to the association between nutritional status and quality of life.

When reviewing the current literature, it is evident that the relationship of nutritional status with quality of life is generally viewed as a one-dimensional issue [21, 22]. While some studies explore the association of malnutrition with quality of life [22], others focus solely on its connection with frailty or depression [18, 23]. However, in clinical practice, these syndromes are rarely seen in isolation and often interact in complex ways. Despite these established relationships, it remains unclear whether nutritional status is independently associated with quality of life when the collective associations of depression, loneliness, and physical frailty are concurrently considered. When the predictive power of strong factors such as depression, loneliness, and frailty is controlled, the evidence regarding the independent predictive role of nutritional status with quality of life is still limited. Addressing this gap is of paramount importance, as determining the independent association of nutrition status holds substantial significance for clinical interventions. In older adults, the perception of loneliness is not merely a concomitant of depression; it is a factor that is associated with appetite and nutritional quality as social networks narrow. The loss of social dining experiences increases the risk of malnutrition by reducing dietary variety, which in turn is linked to a reduction in an individual’s sense of overall well-being, independent of depressive symptoms. Therefore, disentangling the respective associations of loneliness and depression with quality of life is critical for the development of more targeted geriatric interventions. Identifying the unique contribution of nutritional status, apart from these burdens, is crucial for developing comprehensive intervention strategies. Therefore, this study aims to assess the nutritional status of community-dwelling older adults and to examine the independent association of nutritional status with quality of life, while uniquely controlling for the concurrent burdens of depression, frailty, and perceptions of loneliness.

## Methods

This cross-sectional study was conducted between November 2025 and January 2026 and involved 758 volunteer participants aged 65 and over, comprising 330 men and 428 women, living in the province of Ankara. Participants were recruited using a non-probability convenience sampling method. To ensure diversity within the sample, researchers established direct contact with potential participants in various public settings, including parks and local neighborhoods across different districts of Ankara. To mitigate potential selection bias, data collection was conducted in regions representing a wide range of socio-economic backgrounds and various community centers throughout the city. During the recruitment phase, a total of 1,546 older adults were approached, of whom 758 voluntarily agreed to participate in the study, resulting in a response rate of 49%. The study’s sample size was determined using G*Power 3.1 software to ensure adequate statistical power. According to the software programme, for a design with a significance level of α = 0.05, a power (1-β) of 0.95, and a medium effect size (f2 = 0.15), a minimum of 172 participants were required. Separately, to ensure the representativeness of the population of individuals aged 65 and over in Ankara, a 95% confidence interval and a 5% margin of error, the minimum sample size required to represent the population was 384. The study was completed with 758 volunteer participants, exceeding both requirements and providing high statistical power. Data integrity was maintained using a complete-case analysis approach, justified by a final sample size (*n* = 758) that significantly exceeded power analysis requirements. The study included individuals aged 65 and over who were community-dwelling (living at home), were able to read, understand, and communicate in Turkish, were informed about the study, and signed the voluntary participation consent form. Participant eligibility regarding the exclusion criteria was determined through a brief preliminary assessment and health declaration during face-to-face interviews. Individuals with a known cognitive or neuropsychological disorder (such as a diagnosis of dementia), those who failed to correctly answer basic orientation questions regarding time, place, and person during the initial interview, individuals with severe mental illness -such as schizophrenia, bipolar disorder, or major depression- or those under active psychiatric treatment, as well as those who were bedridden, unable to perform daily living activities independently, or could not comprehend the survey questions due to severe vision or hearing loss, were excluded from the study. Ethical approval for the study was obtained from the University of Health Sciences Gülhane Non-Pharmaceutical and Non-Medical Device Interventional Research Ethics Committee on 20 November 2025, with decision number 2025/034. All participants were informed about the purpose of the study, provided detailed information on confidentiality principles and the basis of voluntariness, and asked to sign an informed consent form after confirming their voluntary participation.

### Data collection form

During data collection, a structured questionnaire prepared by the researchers was administered to participants via face-to-face interviews. These interviews were conducted by trained researchers who had received standardized training on the administration of the study scales to ensure inter-observer consistency. The data were collected in quiet community settings to provide a comfortable and private environment for the interview process. In addition to sections inquiring about sociodemographic characteristics and anthropometric measurements, the questionnaire form included the UCLA Loneliness Scale Short Form, Mini Nutritional Assessment Short Form (MNA-SF), FRAIL Scale, Geriatric Depression Scale (GDS-15), EUROHIS-QOL-8 - World Health Organisation Quality of Life Questionnaire.

### UCLA loneliness scale short form

The UCLA Loneliness Scale was developed to measure individuals’ perceived levels of loneliness. The scale has versions of varying lengths, and the short form (UCLA-3), consisting of three items, was developed by Hughes et al. [24] to facilitate its application in large samples. The scale measures individuals’ subjective assessments of their ‘lack of friends’, ‘feelings of exclusion’, and ‘isolation’. Each item is rated on a three-point Likert scale (‘1 = Hardly ever’, ‘2 = Sometimes’, ‘3 = Often’). The total score on the scale ranges from 3 to 9, with higher scores indicating greater loneliness. The Turkish adaptation of the scale, along with its validity and reliability study, was conducted by Türk [25]. In the present study, the Cronbach’s alpha coefficient for the scale was calculated as 0.812 and McDonald’s ω was 0.814.

### Mini Nutritional Assessment Short Form (MNA-SF)

The Mini Nutritional Assessment screening tool was developed by Guigoz et al. [26] to quickly and reliably assess the nutritional status of older adults. The MNA-SF is a short form of the original scale. The scale consists of 6 items: food intake over the past three months, weight loss, mobility, acute illness/psychological stress, neuropsychological problems, and body mass index (BMI). Each item is scored on a scale of 0–3, and the total score ranges from 0 to 14. Assessment based on total scores is as follows: 12–14 points: normal nutritional status, 8–11 points: at risk of malnutrition, 0–7 points: malnutrition. The Turkish adaptation, validity, and reliability study of the scale was conducted by Sarikaya et al. [27]. In the present study, internal consistency coefficients for the MNA-SF were not reported, as the instrument functions as a multidimensional screening index rather than a unidimensional scale; its items cover diverse clinical and anthropometric domains, which limits the conceptual appropriateness of global internal consistency indices.

### FRAIL scale

The FRAIL Scale is a measurement tool developed by Morley et al. [28] based on the Phenotypic Frailty Model to assess the level of physical frailty in older adults. The scale consists of five components: fatigue, resilience (physical strength), ambulation (mobility), disease burden, and weight loss. Participants are asked about their fatigue level over the past 4 weeks, their ability to climb one flight of stairs without assistance and walk 100 m (one block), the number of chronic diseases they currently have, and their weight loss over the past year. Each item on the scale is scored 0 or 1, and the total score ranges from 0 to 5. Based on the total score, those scoring 0 points are considered healthy (normal); those scoring 1–2 points are considered pre-frail; and those scoring 3–5 points are considered frail. The Turkish adaptation and validity-reliability study of the scale was conducted by Hymabaccus et al. [29]. In the present study, the Cronbach’s alpha coefficient for the scale was calculated as 0.768 and McDonald’s ω was 0.772.

### Geriatric Depression Scale (GDS-15)

The Geriatric Depression Scale (GDS-15) was developed by Yesavage et al. [30] to assess depressive symptoms in older adults. The 15-item short form of the scale (GDS-15) is a shortened and clinically easier-to-use version of the original 30-item form [31]. The GDS-15 measures core depressive symptoms in older adults, such as mood, loss of interest, hopelessness, lack of energy, worthlessness, and satisfaction with life. Each item on the scale is answered with ‘Yes’ or ‘No’. Some items expressing positive mood are reverse-scored. Each depressive response is scored as 1 point, while other responses are scored as 0 points. The total score ranges from 0 to 15, with 0–4 indicating no depression, 5–8 mild depression, 9–11 moderate depression, and 12–15 severe depression. The Turkish adaptation and validity-reliability study of the scale was conducted by Durmaz et al. [32]. In the present study, the Cronbach’s alpha coefficient for the scale was calculated as 0.860 and McDonald’s ω was 0.856.

### EUROHIS-QOL-8 world health organisation quality of life questionnaire-8

EUROHIS-QOL-8 is a shortened form of the WHOQOL-100 and WHOQOL-BREF scales developed by the World Health Organisation (WHO) to assess individuals’ overall quality of life [33]. This 8-item short form, developed by Schmidt et al. [33], comprehensively assesses quality of life in terms of physical, psychological, social, and environmental dimensions. The scale comprises 8 items covering areas such as ‘general satisfaction with life’, ‘health satisfaction’, ‘energy’, ‘maintaining daily activities’, ‘self-satisfaction’, ‘satisfaction with social relationships’, ‘financial resources’, and ‘satisfaction with living conditions’. Each item is rated on a 5-point Likert scale (1 = very poor/not at all satisfied – 5 = very good/completely satisfied). The total score obtained from the scale ranges from 8 to 40, with higher scores indicating a higher quality of life. The Turkish adaptation, validity and reliability study of the scale was conducted by Eser & Lağarlı [34]. In the present study, the Cronbach’s alpha coefficient for the scale was calculated as 0.796 and McDonald’s ω was 0.794.

### Assessment of body weight

Participants’ heights and body weights were measured and recorded by the researchers using standardized portable scales and stadiometers. Body Mass Index (BMI) was used to determine individuals’ body weight status. BMI is calculated by dividing body weight (kg) by height squared (m²). According to the World Health Organisation classification, BMI < 18.5 kg/m² is considered ‘underweight’, 18.5–24.9 kg/m² is ‘normal’, 25.0–29.9 kg/m² is “overweight”, and ≥ 30.0 kg/m² is ‘obese’ [35].

### Statistical analysis

The data obtained from the study were analysed using the IBM SPSS (Statistical Package for the Social Sciences) 26.0 software package. Descriptive data were presented as numbers (n) and percentages (%) for categorical variables and as mean (X̄) and standard deviation (SD) values for continuous variables. Prior to analysis, the normality of continuous variables was assessed using the Kolmogorov-Smirnov test. To account for the sensitivity of the Kolmogorov-Smirnov test in large sample sizes, the normality of the data was further verified through visual diagnostics, including histograms and Q-Q plots, as well as an assessment of skewness and kurtosis coefficients. All coefficients were found to be within the acceptable range of − 1.5 to + 1.5 across all scales, confirming that the data satisfied the underlying assumptions for parametric statistical analysis. One-Way ANOVA was used to compare the means of independent groups. Pearson’s Chi-Square test was used to examine the relationships between categorical variables. An independent Multiple Linear Regression model, using variables selected based on their theoretical importance in the literature (theory-driven) and statistical significance, was established to investigate the determinants of nutritional status, and the magnitudes and directions of the correlation coefficients between continuous variables were visualised using a heatmap with colour intensity. Pearson correlation coefficients were used for the heatmap as the data met normality assumptions; this figure is intended to be descriptive, therefore no correction for multiple comparisons was applied. A three-stage Hierarchical Linear Regression Analysis was applied to examine the associations of independent variables with quality of life. In the hierarchical model, the order of variable entry was established based on theoretical frameworks: Model 1 controlled for demographic factors, Model 2 incorporated core geriatric syndromes (depression, frailty, and loneliness), and Model 3 tested the independent contribution of nutritional status (MNA-SF) to quality of life. Prior to regression analysis, the independent variables were examined for multicollinearity; no multicollinearity was found. Multicollinearity was assessed, and Variance Inflation Factor (VIF) values were found to range between 1.074 and 2.486, indicating no significant concerns regarding collinearity. Additionally, the fundamental assumptions of regression—including normality, linearity, and homoscedasticity—were verified through the inspection of P-P plots and the distribution of residuals. Prior to the analysis, categorical variables were subjected to dummy coding: Gender (0: Female, 1: Male), Living Alone (0: No, 1: Yes), and Income Level (Reference: Low Income). A significance level of *p* < 0.05 was accepted in all statistical analyses.

## Results

Table 1 shows the general characteristics of the individuals participating in the study. 56.5% of the participants (*n* = 428) were female, and 43.5% (*n* = 330) were male. The average age of the individuals participating in the study was 71.1 ± 5.79 years. In terms of marital status, 63.1% (*n* = 478) were married, 29.8% (*n* = 226) were widowed, and 7.1% (*n* = 54) were single. In terms of educational attainment, the highest percentage was 26.9% (*n* = 204) for primary school graduates, followed by 24.1% (*n* = 183) for high school graduates. 56.9% (*n* = 431) of participants were retired, and 68.2% (*n* = 517) had a medium level of economic income. 72.7% (*n* = 551) of individuals lived with their families, and 72.3% (*n* = 548) ate their meals with their families. 34.6% of individuals (*n* = 262) reported feeling lonely. Regarding health and lifestyle habits, 76.8% of individuals (*n* = 582) reported not smoking, and 86.3% (*n* = 654) reported not consuming alcohol. The average body mass index of the individuals was 27.3 ± 4.42 kg/m2. Regarding exercise habits, 60.0% of individuals (*n* = 455) reported not exercising for 150 min per week. Furthermore, 79.0% of individuals (*n* = 599) had a chronic disease, 78.9% (*n* = 598) used medication, and 61.9% (*n* = 469) used more than one medication.

**Table 1:** General Characteristics of the Participants (*n* = 758)

Variables		*n* (%) or X̄±SD
Gender	Male	330 (43.5)
	Female	428 (56.5)
Age		71.1 ± 5.79
Marital Status	Married	478 (63.1)
	Single	54 (7.1)
	Widowed	226 (29.8)
Educational Status	Literate	127 (16.8)
	Primary School	204 (26.9)
	Middle School	95 (12.5)
	High School	183 (24.1)
	Undergraduate	136 (17.9)
	Postgraduate	13 (1.7)
Occupation	Retired	431 (56.9)
	Homemaker	229 (30.2)
	Unemployed	63 (8.3)
	Employed	35 (4.6)
Income Level	Low	124 (16.4)
	Moderate	517 (68.2)
	High	117 (15.4)
Living Status	Alone	169 (22.3)
	With Family	551 (72.7)
	Other (caregiver, sibling, relative)	38 (5.0)
Eating Companions	Alone	184 (24.3)
	With Family	548 (72.3)
	With Friends	26 (3.4)
Smoking Status	Yes	176 (23.2)
	No	582 (76.8)
Alcohol Consumption	Yes	104 (13.7)
	No	654 (86.3)
BMI		27.3 ± 4.42
Physical Activity (150 min/week)	Yes	303 (40.0)
	No	455 (60.0)
Chronic Disease	Yes	599 (79.0)
	No	159 (21.0)
Medication Use	Yes	598 (78.9)
	No	160 (21.1)
Polypharmacy	Yes	469 (61.9)
	No	289 (38.1)

*BMI* Body mass index

Table 2 presents the individuals’ scale scores. According to the MNA-SF scale, 34.7% (*n* = 263) of the individuals were at risk of malnutrition, and 7.3% (*n* = 55) were malnourished. According to the UCLA Loneliness Scale, 34.6% of individuals (*n* = 262) reported feeling lonely. When examining frailty status, 40.4% of participants (*n* = 306) were pre-frail, and 15.3% (*n* = 116) were frail. Looking at geriatric depression levels, 27.4% of individuals (*n* = 208) were found to have mild depression and 22.2% (*n* = 168) had moderate depression. The average scores obtained by participants on the scales were 11.5 ± 2.43 for the MNA-SF, 4.9 ± 1.81 for the UCLA Loneliness Scale, 1.1 ± 1.24 for the FRAIL Scale, 5.2 ± 4.03 for the Geriatric Depression Scale, and 27.6 ± 6.27 for the EUROHIS-QOL-8.

**Table 2:** Assessments of the Scales (*n* = 758)

		*n* (%) or X̄±SD
MNA-SF	Normal nutritional status (12–14)	440 (58.0)
	At risk of malnutrition (8–11)	263 (34.7)
	Malnourished (0–7)	55 (7.3)
UCLA	Not lonely (3–5)	496 (65.4)
	Lonely (6–9)	262 (34.6)
FRAIL	Normal (0)	336 (44.3)
	Pre-frail (1–2)	306 (40.4)
	Frail (3–5)	116 (15.3)
GDS-15	No depression (0–4)	381 (50.3)
	Mild depression (5–8)	208 (27.4)
	Moderate depression (9–11)	168 (22.2)
	Severe depression (12–15)	1 (0.1)
MNA-SF Score		11.5 ± 2.43
UCLA Score		4.9 ± 1.81
FRAIL Score		1.1 ± 1.24
GDS-15 Score		5.2 ± 4.03
EUROHIS-QOL-8 Score		27.6 ± 6.27

*MNA-SF* Mini Nutritional Assessment-Short Form, *UCLA* University of California, Los Angeles Loneliness Scale, *FRAIL* Fatigue, Resistance, Ambulation, Illnesses, and Loss of weight scale, *GDS* Geriatric Depression Scale, *EUROHIS-QOL* European Health Interview Survey - Quality of Life

Table 3 shows the general characteristics of individuals according to their malnutrition status. Individuals were divided into three groups: normal nutritional status (*n* = 440), at risk of malnutrition (*n* = 263), and malnourished (*n* = 55). The mean ages of the groups were 70.3 ± 5.37, 71.6 ± 5.44, and 78.8 ± 7.98 years, respectively, and the differences between the groups were statistically significant (*p* < 0.05). The distribution of variables such as gender (*p* = 0.035), marital status, educational status, living arrangements, economic income, and eating habits showed significant differences by malnutrition status (*p* < 0.001). In terms of health status, the presence of chronic disease (*p* = 0.002), exercise, medication use, and polypharmacy were found to be statistically significantly different between malnutrition statuses (p˂0.001). According to the frailty classification, 32.8% (*n* = 38) of frail individuals were malnourished, whereas only 1.2% (*n* = 4) of those in the normal group were malnourished (*p* < 0.001). In geriatric depression categories, 28.0% (*n* = 47) of those with moderate depression were malnourished, while only 0.8% (*n* = 3) of those without depression were malnourished (p˂0.001). When individuals were examined by BMI, the average BMI of individuals in the normal nutritional group was 27.8 ± 4.13 kg/m², that of the group at risk of malnutrition was 27.1 ± 4.60 kg/m², and that of the malnourished group was 23.7 ± 4.15 kg/m². (*p* = 0.032). Post hoc analyses revealed that the malnourished group was significantly different from the other groups. Looking at the scale scores, the UCLA Loneliness score was 4.2 ± 1.43 in the group with normal nutritional status, 5.5 ± 1.82 in the group at risk of malnutrition, and 6.9 ± 1.75 in the malnourished group (p˂0.001). The mean scores for the Frailty, Geriatric Depression, and EUROHIS-QOL-8 scores were significantly different between groups (*p* < 0.001). In the post hoc analyses, all three groups were found to differ significantly from each other on all these scales (*p* < 0.05).

**Table 3:** General Characteristics of the Participants According to Nutritional Status (*n* = 758)

	Normal nutritional status (*n* = 440)	At risk of malnutrition (*n* = 263)	Malnourished (*n* = 55)	*p*
Gender				
Male	208 (63.0)	98 (29.7)	24 (7.3)	0.035
Female	232 (54.2)	165 (38.6)	31 (7.2)	
Age	70.3 ± 5.37^a^	71.6 ± 5.44^b^	78.8 ± 7.98^c^	0.002
Marital Status				
Married	323 (67.6)	135 (28.2)	20 (4.2)	< 0.001
Single	20 (37.0)	28 (51.9)	6 (11.1)	
Widowed	97 (42.9)	100 (44.2)	29 (12.8)	
Educational Status				
Literate	43 (33.9)	56 (44.1)	28 (22.0)	< 0.001
Primary school	127 (62.3)	68 (33.3)	9 (4.4)	
Middle school	58 (61.1)	34 (35.8)	3 (3.2)	
High school	111 (60.7)	68 (37.2)	4 (2.2)	
Undergraduate	93 (68.4)	34 (25.0)	9 (6.6)	
Postgraduate	8 (61.5)	3 (23.1)	2 (15.4)	
Living Status				
Alone	66 (39.1)	84 (49.7)	19 (11.2)	< 0.001
With family	360 (65.3)	165 (29.9)	26 (4.7)	
Other (caregiver, sibling, relative)	14 (36.8)	14 (36.8)	10 (26.4)	
Income Level				
Low	40 (32.3)	61 (49.2)	23 (18.5)	< 0.001
Moderate	321 (62.1)	170 (32.9)	26 (5.0)	
High	79 (67.5)	32 (27.4)	6 (5.1)	
Physical Activity (150 min/week)				
Yes	213 (70.3)	78 (25.7)	12 (4.0)	< 0.001
No	227 (49.9)	185 (40.7)	43 (9.5)	
Chronic Diseases				
Yes	330 (55.1)	218 (36.4)	51 (8.5)	0.002
No	110 (69.2)	45 (28.3)	4 (2.5)	
Medication Use				
Yes	327 (54.7)	221 (37.0)	50 (8.4)	0.001
No	113 (70.6)	42 (26.3)	5 (3.1)	
Polypharmacy				
Yes	246 (52.5)	178 (38.0)	45 (9.6)	< 0.001
No	194 (67.1)	85 (29.4)	10 (3.5)	
Eating Companions				
Alone	73 (39.7)	85 (46.2)	26 (14.1)	< 0.001
With family	352 (64.2)	167 (30.5)	29 (5.3)	
With friends	15 (57.7)	11 (42.3)	0 (0.0)	
Frailty				
Normal	256 (76.2)	76 (22.6)	4 (1.2)	< 0.001
Pre-frail	168 (54.9)	125 (40.8)	13 (4.2)	
Frail	16 (13.8)	62 (53.4)	38 (32.8)	
GDS-15				
No depression (0–4)	301 (79.0)	77 (20.2)	3 (0.8)	< 0.001
Mild depression (5–8)	106 (51.0)	97 (46.6)	5 (2.4)	
Moderate depression (9–11)	33 (19.6)	88 (52.4)	47 (28.0)	
Severe depression (12–15)	0 (0.0)	1 (100.0)	0 (0.0)	
BMI	27.8 ± 4.13^a^	27.1 ± 4.60^a^	23.7 ± 4.15^b^	0.032
UCLA Score	4.2 ± 1.43^a^	5.5 ± 1.82^b^	6.9 ± 1.75^c^	< 0.001
FRAIL Score	0.6 ± 0.85^a^	1.4 ± 1.25^b^	3.0 ± 1.39^c^	< 0.001
GDS-15 Score	3.5 ± 2.99^a^	6.9 ± 3.78^b^	11.2 ± 3.40^c^	< 0.001
EUROHIS-QOL-8 Score	30.2 ± 4.83^a^	24.9 ± 5.84^b^	19.3 ± 6.11^c^	< 0.001

Pearson’s chi-square test, ANOVA (Bonferroni-adjusted multiple comparisons for categorical data and Tukey’s HSD test for continuous data) Different superscript letters (a, b, c) within a row indicate statistically significant differences between groups based on post-hoc tests

*GDS-15* Geriatric Depression Scale-15, *BMI* Body mass index, *UCLA* University of California, Los Angeles Loneliness Scale, *FRAIL* Fatigue, Resistance, Ambulation, Illnesses, and Loss of weight scale, *EUROHIS-QOL-8* European Health Interview Survey - Quality of Life-8

**Fig. 1:**
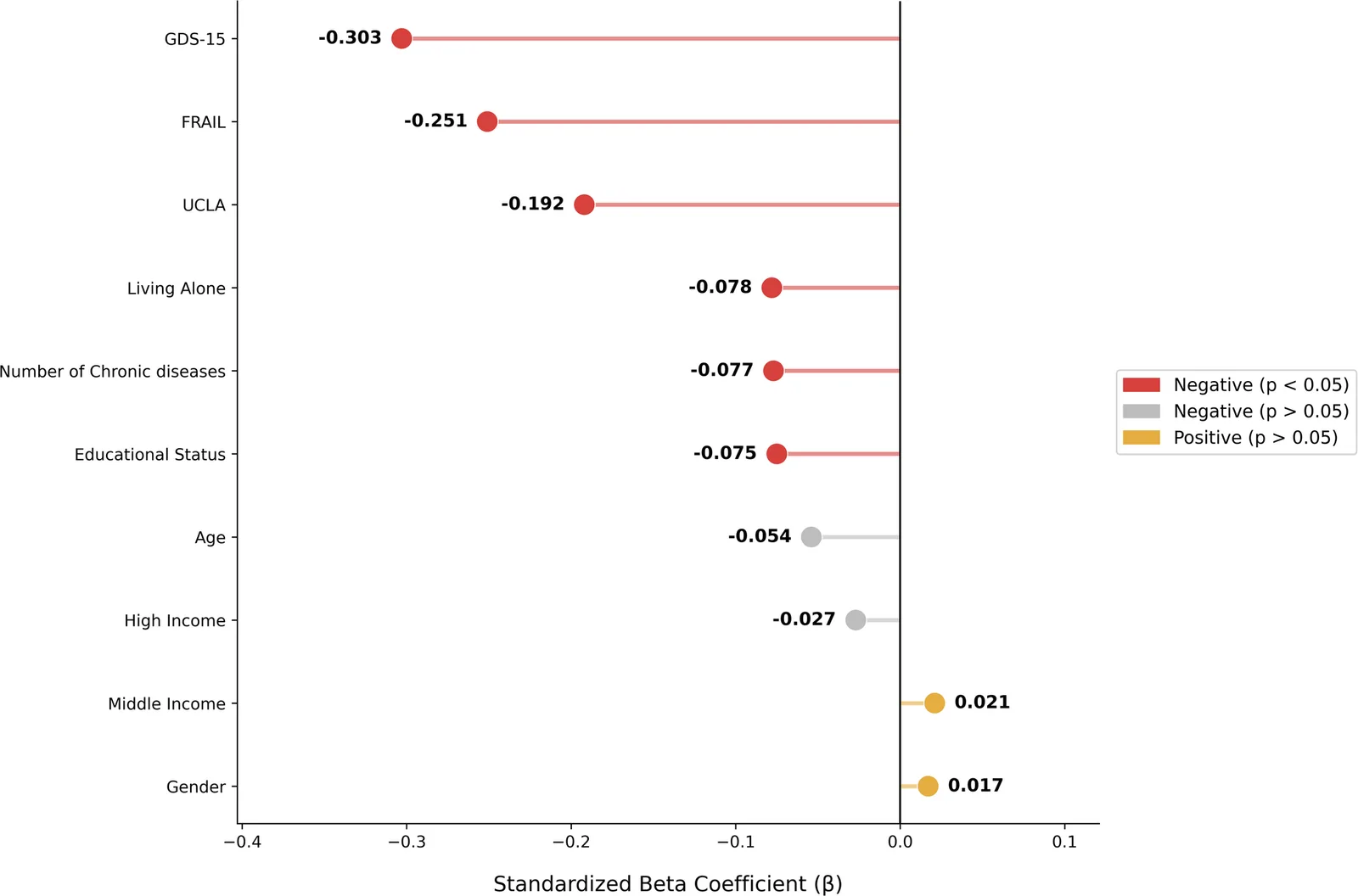
Standardized Coefficients of Predictors for Nutritional Status (MNA-SF) GDS-15: Geriatric Depression Scale-15, FRAIL: Fatigue, Resistance, Ambulation, Illnesses, and Loss of weight scale, UCLA: University of California, Los Angeles Loneliness Scale

Figure 1 shows the standardized coefficients of the factors predicting nutritional status (MNA-SF). According to this, the Geriatric Depression Scale (GDS-15) (β=-0.303), FRAIL Scale (β=-0.251), UCLA Loneliness Scale (β=-0.192), living alone (β=-0.078), number of chronic diseases (β=-0.077) and educational status (β=-0.075) were found to be statistically significant predictors (*p* < 0.05). The association of age, high income, middle income, and gender with nutritional status were not statistically significant (*p* > 0.05).

**Fig. 2:**
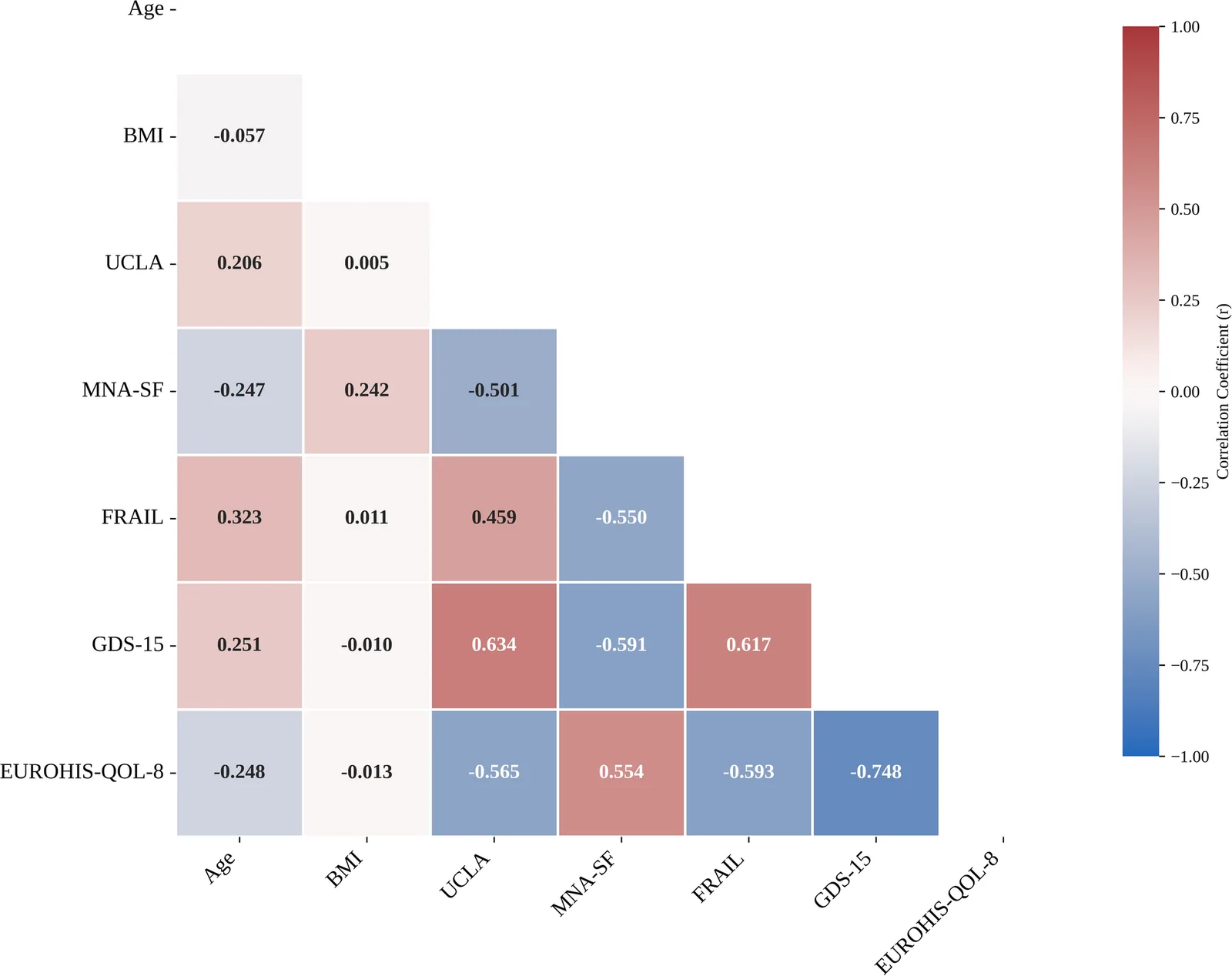
Correlation Heatmap Among Study Variables Note: BMI was included in the correlation matrix for descriptive purposes but was excluded from the multivariable regression model predicting MNA-SF BMI: Body mass index, UCLA: University of California, Los Angeles Loneliness Scale, MNA-SF: Mini Nutritional Assessment-Short Form, FRAIL: Fatigue, Resistance, Ambulation, Illnesses, and Loss of weight scale, GDS-15: Geriatric Depression Scale-15, EUROHIS-QOL-8: European Health Interview Survey-Quality of Life-8

Figure 2 shows the correlation coefficients for the variables in a heat map. The relationships between the variables were evaluated using correlation analysis (*n* = 758). According to the analysis results, the strongest correlation was found to be negative between the Geriatric Depression Scale (GDS-15) and quality of life (EUROHIS-QOL-8) (*r*=-0.748). Positive correlations were observed between age and frailty (*r* = 0.323) and between GDS-15 and loneliness (UCLA) (*r* = 0.634). A negative correlation was found between frailty and quality of life (EUROHIS-QOL-8) (*r*=-0.593); and a positive correlation was found between frailty and depression (GDS-15) (*r* = 0.617). Nutritional status (MNA-SF) showed the highest correlation with the variables frailty (*r*=-0.550) and GDS-15 (*r*=-0.591), both in a negative direction, and with EUROHIS-QOL-8 (*r* = 0.554) in a positive direction.

A three-stage hierarchical regression analysis was conducted to identify the factors associated with individuals’ quality of life (Table 4). Before the analysis, multicollinearity was checked, and no multicollinearity was detected.

**Table 4:** Hierarchical Regression Analysis of Factors Associated with Quality of Life (*n* = 758)

		Model 1				Model 2				Model 3			
	Variables	B (SE)	*β*	t	95% CI	B (SE)	*β*	t	95% CI	B (SE)	*β*	t	95% CI
1	(Constant)	30.084 (3.074)	-	9.787***	24.047–36.121	33.641 (2.290)	-	14.691***	29.144–38.138	28.887 (2.683)	-	10.766***	23.617–34.157
	Gender	0.901 (0.466)	0.070	1.935	-0.013-1.816	0.806 (0.337)	0.063*	2.392	0.144–1.468	0.782 (0.334)	0.061*	2.338*	0.125–1.438
	Age	-0.115 (0.039)	-0.109**	-2.936	-0.192 – -0.038	-0.09 (0.029)	-0.008	-0.295	-0.066-0.048	-0.002 (0.029)	-0.002	-0.072	0.059 − 0.055
	Education Status	0.745 (0.156)	0.183***	4.768	0.438–1.051	-0.042 (0.117)	-0.010	-0.361	-0.273-0.188	-0.007 (0.117)	-0.002	-0.064	-0.237-0.222
	Income Level: Middle ^a^	3.956 (0.609)	0.292***	6.497	2.760–5.152	1.415 (0.457)	0.104**	3.095	0.517–2.313	1.383 (0.453)	0.102**	3.051	0.493–2.273
	Income Level: High ^a^	6.539 (0.845)	0.342***	7.743	4.880–8.197	3.470 (0.627)	0.182***	5.532	2.238–4.702	3.529 (0.622)	0.185***	5.670	2.306–4.751
	Living Alone	-1.368 (0.541)	-0.093*	-2.529	-2.431 – -0.305	0.013 (0.399)	0.001	0.033	-0.770-0.796	0.144 (0.397)	0.010	0.363	-0.636-0.924
	Number of Chronic Diseases	-0.820 (0.183)	-0.164***	-4.473	-1.179 – -0.460	-0.266 (0.136)	-0.053	-1.954	-0.533-0.001	-0.223 (0.136)	-0.045	-1.641	-0.489-0.044
2	Frailty (FRAIL)					-0.791 (0.168)	-0.163***	-4.706	-1.121– -0.461	-0.652 (0.172)	-0.134***	-3.793	-0.989– -0.314
	Loneliness (UCLA)					-0.406 (0.117)	-0.122**	-3.474	-0.636– -0.177	-0.334 (0.118)	-0.100**	-2.826	-0.566– -0.102
	Depression (GDS-15)					-0.790 (0.059)	-0.515***	-13.372	-0.906– -0.674	-0.737 (0.061)	-0.481***	-12.144	-0.856– -0.618
3	MNA-SF									0.284 (0.085)	0.114**	3.326	0.116–0.452
	*R* ^***2***^	0.268				0.622				0.629			
	Adj. *R*^***2***^	0.260				0.616				0.622			
	Δ *F*	31.022***				183.719***				11.059**			
	Δ *R*^***2***^	0.268				0.354				0.007			

*B* Unstandardized regression coefficient, * SE* Standard Error, *β* Standardized regression coefficient, *FRAIL* Fatigue, Resistance, Ambulation, Illnesses, and Loss of weight scale, *UCLA* University of California, Los Angeles Loneliness Scale, *GDS-15* Geriatric Depression Scale-15

^a^ low income as reference

*p < 0.05, **p < 0.01, ***p < 0.001

In Model 1, demographic and health-related variables (gender, age, educational status, economic status, and number of illnesses) were included as control variables, and these variables collectively explained 26.8% of the variance in quality of life (ΔF = 31.022, *p* < 0.001).

Frailty, loneliness, and depression variables were added to Model 2, and the explained variance increased by 35.4% (ΔF = 183.719, *p* < 0.001). The model’s explanatory power increased to 62.2%. In Model 2, after adjusting for the aforementioned demographic factors, frailty (β=-0.163, *p* < 0.001), loneliness (β=-0.122, *p* < 0.01), and depression (β=-0.515, *p* < 0.001) significantly predicted quality of life negatively.

Nutritional status (MNA-SF) was added to Model 3, increasing the explained variance by 0.7% (ΔF = 11.059, *p* < 0.01). The model’s explanatory power increased to 62.9%. In Model 3, while the associations of demographic and psychosocial variables were maintained, the MNA-SF score was observed to significantly predict quality of life independently (β = 0.114, *p* < 0.01).

## Discussion

This study aimed to assess the nutritional status of community-dwelling older adults and to examine the multidimensional relationship between nutritional status and perceived loneliness, frailty, depression, and quality of life. The study’s findings show that although a large proportion of older adults have normal nutritional status, nutritional status is significantly associated with quality of life, even when strong determinants such as age, disease burden, and depression are controlled for. This result suggests that nutritional well-being plays a notable role in maintaining quality of life in older adults.

A meta-analysis evaluating community-dwelling older adults aged 65 and older across 38 countries reported that the prevalence of malnutrition ranged from 0.8% to 24.6% [4]. In our country, studies evaluating nutritional status using the MNA found the prevalence of malnutrition and risk of malnutrition to be 26.9% [7] and 35.2% [36], respectively; while in a study where nutritional status was assessed using GLIM criteria, it was determined to be 24.5% [6]. The prevalence of malnutrition in the community-dwelling older adult population may vary across regions and depending on the screening tools used [5]. These disparities in prevalence rates may be attributed to the varying sensitivities of assessment tools, such as the MNA and GLIM criteria, in detecting malnutrition risk relative to clinical diagnosis. The study found that 42% of participants were at risk of malnutrition or were malnourished. However, the prevalence of malnutrition is related to various factor, including the individual’s physiological and biological characteristics, as well as their social and economic environment [37, 38]. This might be due to the high rates of chronic disease (79.0%) and polypharmacy (61.9%) in our sample. Indeed, our data support this connection, as the rate of normal nutritional status was lower in individuals with chronic diseases (55.1%) compared to those without (69.2%), suggesting that such physiological burdens likely contributed to the observed prevalence. In a study in which 96.6% of participants had at least one chronic disease, the prevalence of malnutrition, assessed using the MNA-SF, was 52.8% [39]. The adverse effects of chronic disease burden and polypharmacy on appetite mechanisms and food intake are well documented in the literature [8]. The findings from this study also support that this physiological burden worsens nutritional status.

One of the most striking results of the study is the relationship between depression and nutritional status. The fact that depression scores are the strongest predictor of MNA-SF scores highlights the significant relationship of depression with nutritional status in the geriatric population. The negative and significant relationship between MNA-SF scores and depression indicates that as depression increases, the risk of malnutrition also increases. While our cross-sectional design prevents establishing a temporal sequence, we hypothesize a potential bidirectional relationship where inadequate nutrition may also impair mood, a link that warrants further investigation through longitudinal studies. The literature also demonstrates that depression is associated with nutritional status [16]. It has been shown that anhedonia and apathy, particularly when accompanied by depressive mood, reduce energy intake in older individuals, and are associated with a higher risk of malnutrition [9, 36]. This relationship may be further intensified by shared mechanistic pathways such as chronic low-grade inflammation and the ‘anorexia of aging’ [9, 16]. Notably, depression scores significantly predicted MNA-SF scores despite half of the participants lacking clinical depressive symptoms. This suggests that even subclinical symptoms may be negatively associated with nutritional status. These results indicate that even mild depressive symptoms may be linked to changes in food intake and poorer nutritional status. Therefore, assessing nutritional status alongside mental health in older adults could be beneficial, as even mild depressive symptoms may represent a relevant factor in the context of malnutrition risk.

The study found a significant negative relationship between frailty and MNA-SF scores and revealed that frailty is one of the essential factors predicting MNA-SF scores. These findings are considered consistent with the literature. A complex, bidirectional relationship between frailty and malnutrition has been reported [10, 11, 40]. Studies have shown that malnutrition is more prevalent in frail older adults and that factors contributing to frailty, such as physical inactivity, depression, and social isolation, further negatively relate to nutritional status [10, 12, 13, 19]. Furthermore, inadequate nutrition may be associated with an accelerated the frailty process by increasing muscle mass loss. These vicious cycles increase the risk of falls and mortality in older individuals. Therefore, it is essential to assess the nutritional and frailty status of community-dwelling older adults [15]. In this study, while the majority of individuals with normal nutritional status (58.1%, *n* = 335/440) did not exhibit frailty, the majority of individuals at risk of malnutrition and those with malnutrition (74.8%, *n* = 238/318) were found to be physically vulnerable, comprising 43.4% (*n* = 138) pre-frail and 31.4% (*n* = 100) frail individuals. This high prevalence of physical vulnerability among those with poor nutritional status highlights a significant clinical overlap between these two conditions in community-dwelling older adults. The fact that more than half of the individuals were physically inactive and used polypharmacy may have contributed to these results.

The study revealed a significant negative correlation between participants’ perception of loneliness and their MNA-SF scores, and that loneliness scale scores significantly predicted MNA-SF scores. This indicates that as perceived loneliness increases, nutritional status deteriorates, highlighting the importance of social connectedness and perceived loneliness for nutritional status in older individuals. It has been shown that people tend to consume more variety and quantity when eating with others, and that factors such as insufficient social interaction or low perceived social support can increase individuals’ feelings of loneliness, which can negatively relate to appetite and food intake [17, 41, 42]. Mikami et al. [17] found that older adults who eat alone are 1.75 times more likely to have poor appetite. Another study found that 37% of older adults who felt lonely two or more days a week were at high risk of malnutrition [43]. The findings from this study support the literature, indicating that perceived loneliness is a factor that should be considered in nutritional screening in the geriatric population, and that social support interventions may significantly improve nutritional status.

The study indicates that the number of illnesses negatively predicts the MNA-SF score and that while 69.2% of individuals without chronic illness have a normal nutritional status, this rate drops to 55.1% in those with chronic illness, demonstrating the adverse effects of chronic disease burden on the risk of malnutrition. These findings are consistent with the literature, which reports that multiple chronic diseases are negatively associated with food intake and nutritional status through increased metabolic requirements, changes in appetite, and functional limitations [44, 45]. It has been shown that the risk of malnutrition is significantly higher in individuals with chronic disease compared to those without, and that the presence of multiple chronic diseases further increases this risk [9]. The results obtained from this study support the need to evaluate chronic disease status and management in the geriatric population in conjunction with nutritional status assessment.

Hierarchical regression analysis, applied to identify factors associated with quality of life, indicated the independent association of nutritional status with quality of life. The fact that demographic and health variables explain 26.8% of the variance in quality of life indicates that age, socioeconomic status, educational level, and chronic disease burden are key determinants of quality of life in the geriatric population. This finding is consistent with previous literature [2, 3, 46] and supports the fundamental association between sociodemographic factors and quality of life.

The increase in explained variance to 62.2% when psychosocial variables such as loneliness, frailty, and depression were added to the regression model demonstrates the importance of psychosocial factors in understanding quality of life. The strategic selection of these three variables (depression, frailty, and loneliness) as primary controls is grounded in their established role as the most dominant psychosocial correlates of Quality of Life (QoL) in older adults, often co-occurring with malnutrition [19, 23]. This is consistent with recent studies showing that quality of life in older adults is related to mental health and social connectedness [18, 19, 23]. Furthermore, the fact that depression emerged as the strongest predictor in the model (β=-0.515) supports the significant association between mental health and the quality of life of older adults. Depressive symptoms are known to be associated with poorer quality of life through energy loss, low motivation, and social withdrawal [20, 36]. A one-year follow-up study conducted with individuals aged 65 and over revealed that depression is one of the most critical factors determining quality of life and that this association persists over time [18]. Similarly, a one-year follow-up study conducted with 945 older adults showed that GDS-15 scores were negatively associated with quality of life [47]. Furthermore, the literature indicates that the negative association between depression and quality of life is more pronounced in older adults [2]. Therefore, improving psychosocial factors is crucial for enhancing the quality of life in this population. The hierarchical entry of these variables allowed for the systematic isolation of these powerful psychosocial “giants” before assessing the unique contribution of nutrition.

Similarly, the fact that frailty (β=-0.163) and perceived loneliness (β=-0.122) also negatively predict quality of life indicates that these factors are adversely associated with not only nutritional status but also overall quality of life. Frailty is negatively associated with the quality of life of older individuals. This is not merely a physical decline but a complex process that has devastating consequences on an individual’s life satisfaction and social functioning [48]. Physical limitations arising from frailty weaken the individual’s sense of self-sufficiency, reducing their enjoyment of life and sense of usefulness [20, 36]. Components of frailty, such as muscle strength loss, fatigue, low physical activity, and slow walking speed can be negatively associated with quality of life by limiting daily living activities. A study of 235 frail/pre-frail older adults found that fatigue and exhaustion lasting more than 5 days per week significantly reduced quality of life scores (EQ-5D-5 L) [23]. Rocha et al. [14] revealed that frailty is the most fundamental predictor of vulnerability, impairing quality of life. It is noted that frailty is a potentially reversible process and that physical exercise and nutritional interventions are the most effective ways to reduce frailty and improve quality of life [23, 48, 49].

In addition to frailty, the perception of loneliness has also been found to be negatively associated with quality of life. This finding is consistent with studies emphasising that social communication is one of the fundamental requirements for health and well-being [18, 19, 47]. Ko et al. [18] found, in a 1-year follow-up study, that the quality of life of older adults who felt lonely declined further over time. Similarly, in a 1-year follow-up study of 945 older adults, social support was positively associated with quality of life, while loneliness was negatively associated [47]. Loneliness and social isolation are closely linked to a decline in the quality of life of older individuals. It is suggested that social isolation can undermine an individual’s sense of social participation and connection, thereby shaking their perception of general well-being and life satisfaction [19]. Similarly, it is stated that a decrease in social contact may be associated with a more rapid decline in cognitive functions due to a lack of mental stimulation, further deteriorating quality of life [16, 19].

One of the key findings of the study is that nutritional status was found to be a significant independent correlate of quality of life even after controlling for demographic, health, and psychosocial variables. Although the unique contribution of nutritional status to the overall variance in quality of life was modest in magnitude, its persistence alongside powerful predictors like depression and frailty underscores nutrition status as an essential and distinct component of geriatric well-being. While the 0.7% incremental variance attributed to the MNA score may appear statistically modest, this finding is of critical significance; it demonstrates that nutritional status remains a robust independent predictor of quality of life even when dominant factors—such as depression and frailty—are concurrently accounted for in the model. Our results align with existing literature [50, 51], suggesting that nutritional status is not merely a secondary byproduct of psychosocial health. Instead, it serves as a fundamental biological cornerstone that directly bolsters physical resilience and functional independence in older adults. This implies that even marginal improvements in nutrition can translate into clinically meaningful differences in a patient’s daily quality of life. Furthermore, this independent contribution provides theoretical weight to the argument that geriatric intervention strategies should extend beyond psychosocial support, integrating nutritional therapy as an indispensable pillar of a holistic care paradigm. MNA-SF scores have been reported to correlate positively with quality of life [52]. Casals et al. [23] also found that nutritional status independently is associated with quality of life. Malnutrition or risk of malnutrition is associated with lower quality of life through loss of muscle strength, fatigue, and functional dependence [23, 50, 51]. Furthermore, inadequate protein and energy intake are linked to impaired immune function and an increased risk of infection, which, in turn, are associated with higher hospitalization rates and lower quality of life [53, 54]. Furthermore, various micronutrient deficiencies, particularly vitamin B12 and folate, are associated with cognitive impairment and frailty in older adults, and low intake of these nutrients may impair mental health, contributing to lower quality of life and increased risk of cognitive decline [1, 21].

The finding in this study that the relationship between nutrition status and quality of life is independent of psychosocial factors contributes significantly to the literature. This suggests that nutritional status may represent an independent target for interventions aimed at improving quality of life. Furthermore, considering the previously discussed reciprocal relationships between depression-nutrition, frailty-nutrition, and loneliness-nutrition, it has been demonstrated that these factors have a mutually reinforcing interaction. The findings reveal that interventions aimed at improving quality of life in older adults should be evaluated multidimensionally. In the present study, loneliness negatively predicted quality of life independently of depression, suggesting that the role of loneliness relates to social participation and belonging rather than psychological distress alone. Given that loneliness is thought to be associated with cognitive decline through mechanisms distinct from those of depression, our results underscore the importance of integrating social interventions with mental health support. Considering the profound influence of psychosocial factors—such as depression, frailty, and loneliness—interventions targeting these domains appear to be highly beneficial. Furthermore, the independent contribution of nutritional status to quality of life—although more interventional or longitudinal research is required to confirm these associations-substantiates the consideration of nutrition as a potentially significant target for geriatric care. From a clinical perspective, this increase in explanatory power highlights the necessity of integrated interventions; nutritional health should not be viewed as a secondary issue, but rather as an indispensable and independent goal within comprehensive geriatric care models. 

It is crucial to acknowledge that, by design, the MNA-SF incorporates items pertaining to mobility and neuropsychological status, which inherently creates a degree of conceptual overlap with our other primary variables, specifically frailty and depression. Although our multicollinearity diagnostics (VIF values) confirm the absence of statistical redundancy, this inherent intersection suggests that the independent relationship observed between MNA-SF scores and quality of life reflects a comprehensive, multidimensional nutritional profile that extends beyond mere nutrient intake. Ultimately, the contribution of nutritional status can be interpreted as a holistic indicator of geriatric health—one that remains a distinct determinant of well-being even when these overlapping clinical components are concurrently accounted for within the model. This study has certain limitations that warrant consideration. Firstly, due to its cross-sectional design, a definitive cause-and-effect relationship between malnutrition, the psychosocial factors examined, and quality of life cannot be established, and the possibility of reverse causality—whereby poor quality of life is associated with deteriorated nutritional status—cannot be ruled out. Secondly, collecting research data from a single geographical region limits the generalisability of the findings to the broader population. Thirdly, the use of non-probability convenience sampling, coupled with the nature of volunteer recruitment, may introduce a risk of selection bias; participants might represent a segment of the population with higher health consciousness or physical activity levels than the general public. Fourthly, exclusion criteria such as major depression and cognitive impairment were based on participant self-reports and face-to-face preliminary assessments rather than gold-standard clinical diagnostic tests, which may have introduced some degree of sample deviation. While the exclusion of individuals with cognitive impairment was a necessary step to safeguard the accuracy of survey responses and preserve internal validity, it inherently biased the sample toward healthier and more functional individuals compared to the general older adult population. This, in turn, may limit the external validity and the generalization of the findings to the wider, more frail, or cognitively impaired geriatric population. Another limitation is that the data were collected using self-report scales, which may introduce recall bias. Furthermore, the reliance on the MNA-SF as a screening tool is a limitation; such tools cannot fully replace a comprehensive diagnostic assessment that includes biochemical markers or a detailed physical examination. Additionally, the potential conceptual overlap between MNA-SF items and the criteria for frailty and quality of life—and similarly between the GDS-15 and quality of life—may have influenced the strength of the observed correlations. Lastly, although the analysis followed pre-defined hypotheses, the potential for Type I error due to multiple testing remains a conceptual limitation. 

Nevertheless, the study’s most important strength is its holistic approach to malnutrition in conjunction with geriatric syndromes such as depression, frailty, and loneliness. Furthermore, its community-dwelling implementation and large sample size increase the reliability of the results.

## Conclusions

In conclusion, this study used hierarchical regression analysis to suggest that quality of life in older adults has a multifactorial structure and that, despite the dominant association of psychosocial factors, nutritional status is independently associated with quality of life. In light of these findings, these findings suggest that considering comprehensive care models that combine psychosocial support, mental health services, and nutritional interventions may be beneficial to improve quality of life in the geriatric population. Particularly in primary healthcare institutions and community health centres, conducting nutritional status screenings for the geriatric population and integrating frailty assessments, depression screenings, and loneliness assessments into these evaluations could potentially offer opportunities for early intervention. However, further longitudinal and interventional research is necessary to confirm whether such integrated approaches are associated with sustained improvements in the quality of life of older individuals.

## Supplementary Information

Supplementary Material 1.

Supplementary Material 2.

## Data Availability

The data supporting the findings of this study are available from the corresponding authors upon request.

## Abbreviations

MNA-SF: Mini Nutritional Assessment-Short Form
UCLA: University of California, Los Angeles Loneliness Scale
FRAIL: Fatigue, Resistance, Ambulation, Illnesses, and Loss of Weight Scale
GDS-15: Geriatric Depression Scale-15
EUROHIS-QOL-8: European Health Interview Survey - Quality of Life-8
GLIM: Global Leadership Initiative on Malnutrition
BMI: Body Mass Index
