# Supplementary material for: The independent association of nutritional status with quality of life beyond depression, frailty, and loneliness in community-dwelling older adults: a cross-sectional study
Source: BMC Geriatr. 2026 Jul 3;26:1081. doi: 10.1186/s12877-026-07772-5 (PMC13499333; doi:10.1186/s12877-026-07772-5)
Supplement: Supplementary file 1 [file 12877_2026_7772_MOESM1_ESM.docx]

*QoL* = 28.887 + (0.782 x *Gender*) - (0.002 x *Age*) - (0.007 x *Education*) + (1.383 x *Income_Middle_*) + (3.529 x *Income_High_*) + (0.144 x *Living Alone*) - (0.223 x *Chronic Diseases*) - (0.652 x *FRAIL*) - (0.334 x *UCLA*) - (0.737 x *GDS_15_*) + (0.284 x *MNA_SF_*) + ɛ
